# Supplementary material for: Establishment and Characterization of Novel Human Intestinal In Vitro Models for Absorption and First-Pass Metabolism Studies
Source: Int J Mol Sci. 2022 Aug 30;23(17):9861. doi: 10.3390/ijms23179861 (PMC9456142; doi:10.3390/ijms23179861)
Supplement: Supplementary file 1 [file ijms-23-09861-s001.zip › ijms-1859251-supplementary.pdf]

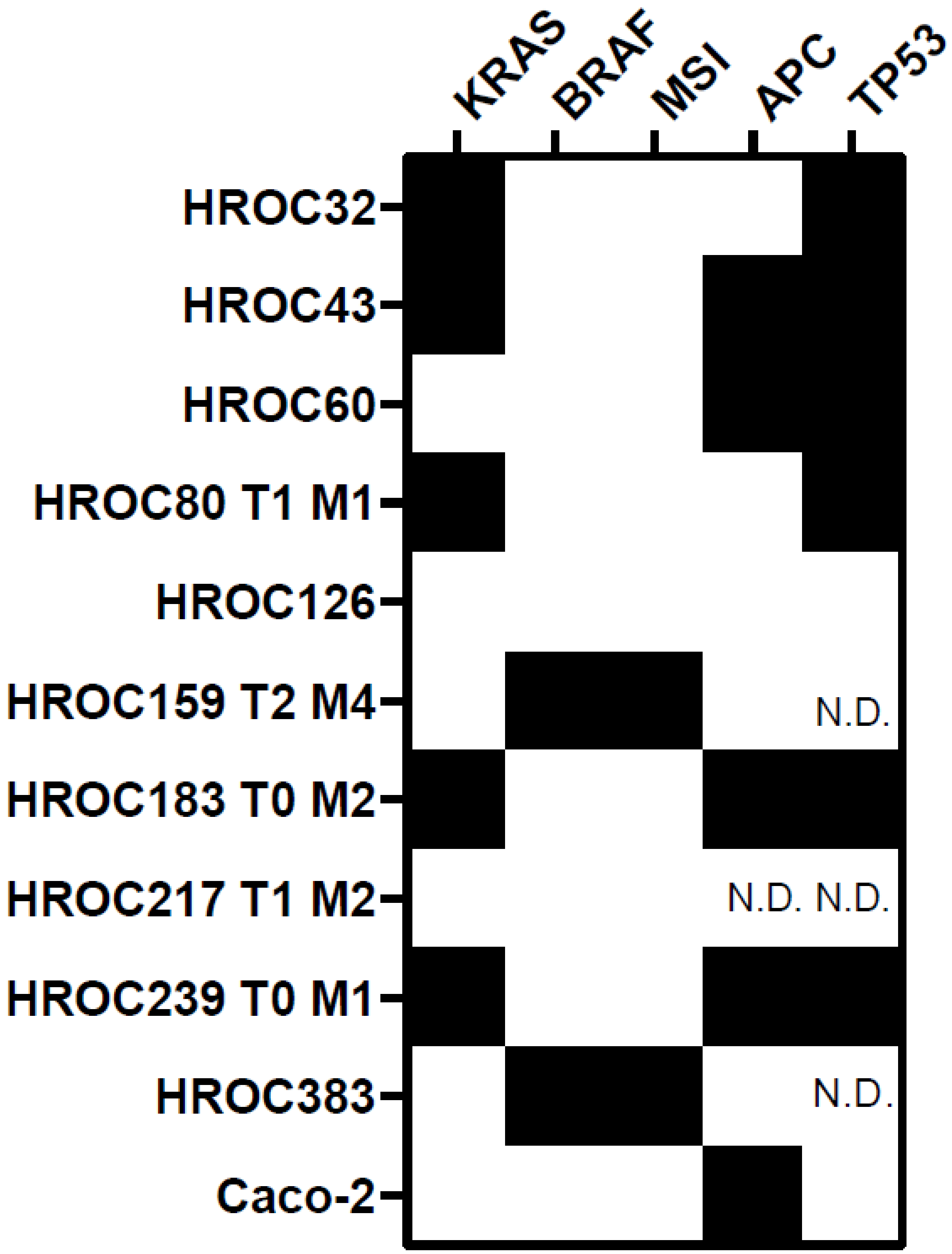

Figure S1. Mutational profile of the HROC cell panel and Caco-2. Heatmap showing somatic cancer driver mutations of HROC and Caco-2 cell lines.

Table S1. Comparison of patient data, morphology assessment, and barrier integrity potential of the HROC 2D cell lines included in this study.

Cell Line	Age, Gender	Localization	$TEER_{max}$ ($\Omega\cdot cm^2$)	Reason for Exclusion
HROC18	65, F	cecum	<100	
HROC24	98, M	ascendens	<300	
HROC24 T1 M1			0	not stable, gap formation
HROC39	69, M	ascendens	<300	
HROC40	69, M	descendens	0	slow proliferation
HROC46 T0 M1	66, M	ascendens	n.d.	dome formation
HROC50 T1 M5	67, F	ascendens	<2000	strong peeling off effect
HROC57	43, M	ascendens	<100	
HROC69	62, M	ascendens	<100	
HROC87 T0 M2	76, F	ascendens	<100	
HROC107	74, M	sigmoid	<100	
HROC113	41, F	ascendens	<300	
HROC131 T0 M3	75, F	ascendens	0	growing in colonies
HROC147 T0 M1	54, M	recto-sigmoid	0	growing in colonies
HROC147Met1			0	growing in colonies
HROC173	45, M	ascendens	<1500	gap formation
HROC212	74, F	cecum	<100	
HROC222 T1 M2	79, M	transversum	<1000	not stable
HROC257	84, F	ascendens	<100	
HROC252Tu2 T2 M2	45, M	sigmoid	<1000	not stable, gap formation
HROC277 T0 M1	77, M	cecum	<100	
HROC277Met1 T0 M2		liver metastasis 1	<1000	no wound healing
HROC277Met2	78, M	liver metastasis 2	<200	
HROC278 T0 M1	76, F	ascendens	0	growing in colonies
HROC278Met T2 M2		peritoneal metastasis	<100	
HROC285 T0 M2	30, F	descendens	<3000	strong peeling off effect
HROC296	92, F	ascendens	<100	
HROC300 T2 M1	73, M	rectum	<1500	no wound healing
HROC300Met1 T3 M4		liver metastasis	<2000	no wound healing
HROC309	86, M	descendens	<100	
HROC315 T1 M2	42, F	descendens	<200	

HROC324	55, F	cecum	<100	
HROC357	41, F	transversum	<300	
HROC370	77, F	cecum	<100	
HROC374	64, M	ascendens	<300	
HROC383 T0 M2	83, F	transversum	<3000	HROC383 in the panel
HROC415Met1 T0 M4	33, M	abdominal wall	<300	
HROC439	73, M	transversum	<2000	not stable, gap formation
HHC6548 T1 M1	26, M	ascendens	<200	

Cells (4×10^4) seeded in 24-well transwell inserts were maintained in full medium at 37 °C and TEER levels were measured daily over a period of 21 days. n.d.: not detected.

Table S2. Genetic features, patient data, and basic characteristics of the final panel of HROC cell lines and Caco-2.

Cell Line	Localization	Age, Gender	UICC	Mol. Type	Cell Migration [in µm/h]	Doubling Time [in h]
HROC32	ascendens	82, F	IV	spSTD	1.8 (±0.2)	59.7 (±6.9)
HROC43	ascendens	72, M	III B	CIMP-L	2.5 (±1.2)	68.4 (±12.2)
HROC60	ascendens	71, M	I	CIMP-L	6.2 (±1.3)	64.5 (±14.6)
HROC80 T1 M1	coecum	72, M	III B	spSTD	1.8 (±1.8)	52.4 (±13)
HROC126	rectum	58, F	III B	spSTD	4.4 (±0.5)	44.5 (±5.7)
HROC159 T2 M4	coecum	78, F	II A	spMSI	4.7 (±2)	59 (±14.2)
HROC183 T0 M2	ascendens	59, F	III C	CIMP-H	2.3 (±1.4)	43.9 (±5.5)
HROC217 T1 M2	ascendens	73, M	III B	spSTD	2.7 (±1.6)	64.6 (±11.6)
HROC239 T0 M1	rectum	72, F	III C	spSTD	0.7 (±0.3)	50.9 (±11.4)
HROC383	transversum	83, F	II A	spMSI	4.5 (±1.4)	58.2 (±13.6)
Caco-2	colon	72, M	-	CIMP-L	7.7 (±1.9)	52.1 (±3.2)

Patient data and characteristics of the HROC cell lines in the final panel. Detailed information can be found in Mullins et al., 2019.
